# Implications for determining the optimal treatment for locally advanced rectal cancer in elderly patients aged 75 years and older

**DOI:** 10.18632/oncotarget.4599

**Published:** 2015-06-23

**Authors:** Jue-feng Wan, Ji Zhu, Gui-chao Li, Wen-jie Sun, Zhen Zhang

**Affiliations:** ^1^ Department of Radiation Oncology, Fudan University Shanghai Cancer Center, Department of Oncology, Shanghai Medical College, Fudan University, Shanghai, China

**Keywords:** rectal cancer, elderly, optimal, SEER

## Abstract

Patients were excluded if they were older than 75 years of age in most clinical trials. Thus, the optimal treatment strategies in elderly patients with locally advanced rectal cancer (LARC) are still controversial. We designed our study to specifically evaluate the cancer specific survival of four subgroups of patients according to four different treatment modalities: surgery only, radiation (RT) only, neoadjuvant RT and adjuvant RT by analyzing the Surveillance, Epidemiology, and End Results (SEER)-registered database. The results showed that the 5-year cancer specific survival (CSS) was 52.1% in surgery only, 27.7% in RT only, 70.4% in neoadjuvant RT and 60.4% in adjuvant RT, which had significant difference in univariate log-rank test (*P* < 0.001) and multivariate Cox regression (*P* < 0.001). Thus, the neoadjuvant RT and surgery may be the optimal treatment pattern in elderly patients, especially for patients who are medically fit for the operation.

## INTRODUCTION

Neoadjuvant chemoradiotherapy (CRT) followed by total mesorectal excision (TME) is a standard treatment in patients aged 75 years and younger with locally advanced rectal cancer (LARC) [1–5]. Patients were excluded if they were older than 75 years of age in most clinical trials. Elderly patients are more likely to have other concomitant chronic illnesses, which may increase the risk of complications and even death during treatment. Thus, the optimal treatment strategies in elderly patients are still controversial.

We designed our study to specifically evaluate the cancer specific survival of four subgroups of patients according to four different treatment modalities: surgery only, radiation (RT) only, neoadjuvant RT and adjuvant RT by analyzing the Surveillance, Epidemiology, and End Results (SEER)-registered database.

## RESULTS

### Patient characteristics

**Figure 1:**
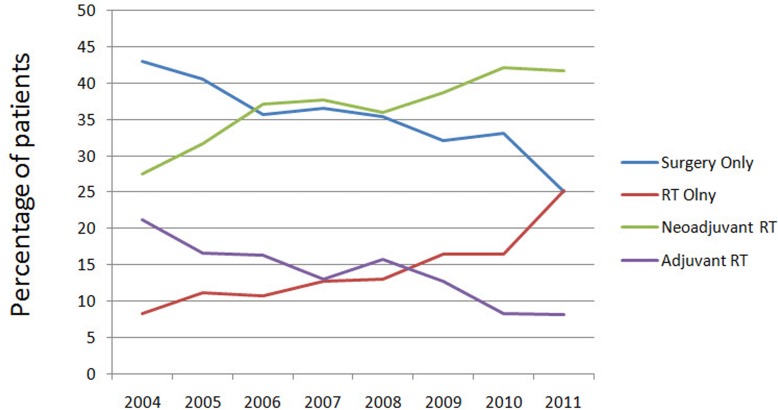
Patterns of care are illustrated for patients who were diagnosed with rectal cancer from 2004 to 2011 according to treatment modality RT indicates radiotherapy.

We identified 4,121 eligible elderly patients with LARC in SEER database during the 8-year study period (between 2004 and 2011), which included 1460 patients in surgery only, 577 patients in RT only, 1498 patients in neoadjuvant RT and 586 patients in adjuvant RT. There were 2077 (50.4%) males and 2044 (49.6%) females. Patient demographics and pathological features are summarized in Table 1. Compared with the decreasing pattern of surgery only and adjuvant RT, the increasing trends of neoadjuvant RT and RT only were observed from 2004 to 2011 (Figure 1).

**Table 1:** Patient characteristics

Variable	Total	Surgery only	RT only	Neoadjuvant RT	Adjuvant RT	P value
	n=4,121	n=1,460	n=577	n=1,498	n=586	
Sex						<0.001
Male	2077	45.8%	48.2%	55.3%	51.4%	
Female	2044	54.2%	51.8%	44.7%	48.6%	
Race						0.359
White	3504	85.8%	82.3%	85.1%	85.5%	
Black	265	5.6%	8.5%	6.6%	6%	
Other	352	8.6%	9.2%	8.3%	8.5%	
Pathological grading						<0.001
Grade I	412	9%	10.9%	9.7%	12.3%	
Grade II	2703	67.9%	62.9%	66.9%	59%	
Grade III	380	10%	8%	8.9%	9.2%	
Grade IV	65	1%	2.9%	1.2%	2.6%	
Unknown	561	12.1%	15.3%	13.2%	16.9%	
Histological Type						<0.001
Adenocarcinoma	3847	93.3%	97.6%	92.3%	92%	
Mucinous/Signet ring cell	274	6.7%	2.4%	7.7%	8%	

### Impact of different treatment strategies on survival outcomes in elderly patients with rectal cancer

**Figure 2:**
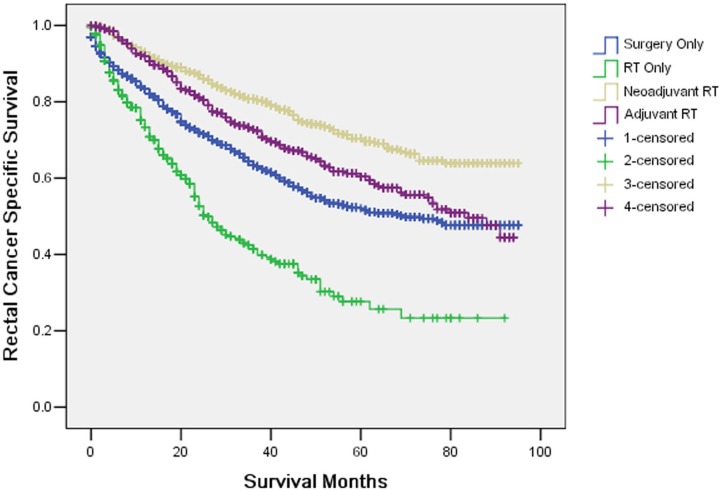
Survival curves in rectal patients according to four subgroups The 5-year cancer specific survival was 52.1% in surgery only, 27.7% in RT only, 70.4% in neoadjuvant RT and 60.4% in adjuvant RT, which had significant difference in univariate log-rank test (*P* < 0.001) and multivariate Cox regression (*P* < 0.001).

The 5-year CSS was 52.1% in surgery only, 27.7% in RT only, 70.4% in neoadjuvant RT and 60.4% in adjuvant RT, which had significant difference in univariate log-rank test (*P* < 0.001) (Figure 2). Besides, mucinous/signet-ring cancer (*P* = 0.022) was identified as a significant risk factor for poor survival on univariate analysis (Table 2). When multivariate analysis with Cox regression was performed, also only treatment patterns and histological type of tumor were the two prognostic factors (Table 2).

**Table 2:** Univariate and multivariate survival analyses of rectal cancer patients according to various clinicopathological variables

Variable	n	5-year CSS (%)	Univariate	Multivariate
			P	P
Sex			0.224	0.601
Male	2077	58.6		
Female	2044	56.9		
Race			0.288	0.259
White	3504	57.2		
Black	265	57.6		
Other	352	63.9		
Treatment pattern			<0.001	<0.001
Surgery only	1460	52.1		
RT only	577	27.7		
Neoadjuvant RT	1498	70.4		
Adjuvant RT	586	60.4		
Histological Type			0.022	0.013
Adenocarcinoma	3847	58		
Mucinous /Signet ring cell	274	53.2		

## DISCUSSION

The incidence of rectal cancer is highest in elderly patients. However, patients aged 75 years and older are often underrepresented in randomized studies. Therefore, it is not clear whether results of rectal cancer studies are equally applicable to elderly patients. Ideally, randomized studies for elderly patients should be performed. Extrapolation of results of younger patients may not be appropriate. In our study, we found elderly patients in neoadjuvant RT had the best CSS.

Higher rates of surgical complications, more prevalent co-morbidities, and poorer performance status limit the standard use of multidisciplinary treatment in older patients, and treatment deviation is higher in elderly patients than younger patients. A Surveillance, Epidemiology, and End Results (SEER)-Medicare study of patients more than 65 years of age who were receiving postoperative therapy for rectal cancer showed that 96.6% of Stage III rectal cancer patients completed radiation therapy, only 68.2% and 67.5% completed chemotherapy and both modalities. Among Stage II cancer patients, 91.5% completed radiation therapy but only 49.8% and 47.6% completed chemotherapy both modalities [9]. The rates of treatment deviation may be even higher among individuals 75 years or older.

Aparicio et al. found a standard cancer treatment according to recommendations was performed in 53 (48%) colorectal cancer patients: adjuvant chemotherapy in 6/23 patients with stage III tumour, palliative chemotherapy in 3/18 patients with stage IV tumour and adjuvant radiotherapy in 4/14 patients who had a rectal tumour resection [10]. Margalit et al. showed 92% rectal patients aged 75 years and older (33/36) completed the planned radiotherapy (RT) dose, 25% (9/36) required an RT-treatment break, 11% (4/36) were hospitalized, and 33% (12/36) had a dose reduction, break, or discontinuation of concurrent chemotherapy [11].

In contrast, Tougeron et al. found in selected elderly patients, chemoradiotherapy is well-tolerated, without any significant increase in adverse events, and the results are similar to those recorded in younger patients [12]. Cai et al. also suggested that although toxicities may be significant, elderly patients with rectal cancer of varied stages can be safely treated with RT or CRT with careful monitoring and frequent modification of treatment [13].

In the Dutch TME-study, patients aged 75 years and older showed a better response in the study arm when compared to younger patients. Younger patients have a significantly lower local recurrence rate of 5.2% after preoperative radiotherapy versus 11% for patients without preoperative radiotherapy (*p* = 0.001). However, overall survival at 5 years, distant metastases free survival and cancer-free survival

were not improved. Whereas in the elderly, apart from local recurrence rate (5.4% versus 14%, *p* = 0.02), also distant metastases free survival (81% versus 69%, *p* = 0.07) and cancer-free survival (81% versus 66%, *p* = 0.03) were improved. Thus, the biological behavior of rectal cancer in the elderly in response to radiotherapy is better than in younger patients [14]. Moreover, no significant differences with respect to postoperative morbidity and mortality were found between neoadjuvant RT and surgery only [14–16]. Therefore, neoadjuvant RT and surgery may be the optimal treatment strategy in elderly patients, especially to patients medically fit for the operation.

Surgery is more effective than radiation only. Thus, it is meaningful to optimize the condition of the patient; making him or her more fit for the operation. All colorectal operations carry significant associated risk. To facilitate the best outcomes it is essential to perform a comprehensive evaluation of patient risk preoperatively. Once risk factors are identified the appropriate steps must be taken to minimize their effects. The evaluation of the patient can be broken down by organ systems such as cardiac, pulmonary, hepatic, renal, and gastrointestinal. Additionally, one can assess whether the patient is at risk for infection, hyperglycemia, malnutrition, venous thromboembolism, and anemia. There are many preemptive steps that can be taken to improve patient outcomes in all of these categories. The ultimate goal is achieving improved outcomes [17–23].

Although this is a large population-based study, it has several potential limitations. First, the SEER registry does not collect comorbidity data. One reason that the elderly patients may be undergoing less aggressive treatment may be due to comorbidities. Second, our study is the lack of data in the SEER registry on the use of chemotherapy, resulting in a potentially significant confounder in the current study. It is possible that

patients may have received adjuvant chemotherapy or preoperative chemotherapy. Because preoperative chemoradiation has been the standard of care for patients with locally advanced rectal cancer (LARC). As for patients who are older than 75 years of age, they may receive less chemotherapy. Finally, the current analysis of the nonrandomized patient population could not exclude the possibility of selection bias. One of the treatment patterns was chose for patients may according to their performance status and comorbidities. However, if patients are medically fit for surgery, they may benefit from neoadjuvant radiation.

In conclusion, multimodal therapy is underused in elderly patients. It appears that standard treatment established in young patients is not applied to every elderly patient. However, the neoadjuvant RT and surgery may be the optimal treatment pattern in elderly patients, especially for patients who are medically fit for the operation.

## MATERIALS AND METHODS

### Patient selection in the SEER database

The SEER, a population-based reporting system, was surveyed for the retrospective collection of data used in the analysis. The SEER program collects and publishes cancer incidence and survival data from 18 population-based cancer registries, covering >25% of the US population. Because no personal identifying information was used in the analysis, this study was granted an exemption from the Institutional Review Board of the study institution on March 30, 2012.

Cases of rectal cancer (C20.9 Rectum, NOS) from 2004 to 2011 were extracted from the SEER database (SEER*Stat 8.1.5) according to the Site Recode classifications with limitation to radiation prior to surgery and radiation preoperatively and post-surgery. Histological type were limited to adenocarcinoma (ICD-03, 8140/3, 8210/3, 8261/3, 8263/3), mucinous adenocarcinoma (ICD-03, 8480/3), and signet ring cell carcinoma (ICD-03, 8490/3). We selected this range because American Joint Committee on Cancer (AJCC) TMN stage was available since 2004. Other exclusion criteria were as follows: synchronous distance metastases and patients with unknown TNM stage.

### Statistical analysis

Age, sex, race, histological grade, histotype and cancer specific survival (CSS) were extracted from SEER database. CSS was calculated from the date of diagnosis to the date of cancer specific death. Deaths attributed to the rectal cancer were treated as events and deaths from other causes were treated as censored observations. The Kaplan-Meier method was used to estimate the CSS [6]. The association between each of the potential prognostic factors and the estimated CSS was tested with the log-rank test [7]. Multivariate analysis was performed using the Cox regression model [8]. The statistical test was two sided and *P* < 0.05 was considered statistically significant. PASW Statistics 13 (SPSS Inc., Chicago, USA) was used for the statistical analysis.

## References

[1] Aschele C, Cionini L, Lonardi S, Pinto C, Cordio S, Rosati G, Artale S, Tagliagambe A, Ambrosini G, Rosetti P, Bonetti A, Negru ME, Tronconi MC, Luppi G, Silvano G, Corsi DC (2011). Primary tumor response to preoperative chemoradiation with or without oxaliplatin in locally advanced rectal cancer: pathologic results of the STAR-01 randomized phase III trial. J Clin Oncol.

[2] Gerard JP, Azria D, Gourgou-Bourgade S, Martel-Laffay I, Hennequin C, Etienne PL, Vendrely V, Francois E, de La Roche G, Bouche O, Mirabel X, Denis B, Mineur L, Berdah JF, Mahe MA, Becouarn Y (2010). Comparison of two neoadjuvant chemoradiotherapy regimens for locally advanced rectal cancer: results of the phase III trial ACCORD 12/0405-Prodige 2. J Clin Oncol.

[3] Rodel C, Liersch T, Becker H, Fietkau R, Hohenberger W, Hothorn T, Graeven U, Arnold D, Lang-Welzenbach M, Raab HR, Sulberg H, Wittekind C, Potapov S, Staib L, Hess C, Weigang-Kohler K (2012). Preoperative chemoradiotherapy and postoperative chemotherapy with fluorouracil and oxaliplatin versus fluorouracil alone in locally advanced rectal cancer: initial results of the German CAO/ARO/AIO-04 randomised phase 3 trial. Lancet Oncol.

[4] Sauer R, Becker H, Hohenberger W, Rodel C, Wittekind C, Fietkau R, Martus P, Tschmelitsch J, Hager E, Hess CF, Karstens JH, Liersch T, Schmidberger H, Raab R (2004). Preoperative versus postoperative chemoradiotherapy for rectal cancer. N Engl J Med.

[5] Sauer R, Liersch T, Merkel S, Fietkau R, Hohenberger W, Hess C, Becker H, Raab HR, Villanueva MT, Witzigmann H, Wittekind C, Beissbarth T, Rodel C (2012). Preoperative versus postoperative chemoradiotherapy for locally advanced rectal cancer: results of the German CAO/ARO/AIO-94 randomized phase III trial after a median follow-up of 11 years. J Clin Oncol.

[6] Kaplan E MP (1958). Nonparametric estimation from incomplete observations. J Am Stat Assoc.

[7] Mantel N (1966). Evaluation of survival data and two new rank order statistics arising in its consideration. Cancer Chemother Rep.

[8] Gill RD (1992). Multistate life-tables and regression models. Math Popul Stud.

[9] Dobie SA, Warren JL, Matthews B, Schwartz D, Baldwin LM, Billingsley K (2008). Survival benefits and trends in use of adjuvant therapy among elderly stage II and III rectal cancer patients in the general population. Cancer.

[10] Aparicio T, Navazesh A, Boutron I, Bouarioua N, Chosidow D, Mion M, Choudat L, Sobhani I, Mentre F, Soule JC (2009). Half of elderly patients routinely treated for colorectal cancer receive a sub-standard treatment. Crit Rev Oncol Hematol.

[11] Margalit DN, Mamon HJ, Ancukiewicz M, Kobayashi W, Ryan DP, Blaszkowsky LS, Clark J, Willett CG, Hong TS (2011). Tolerability of combined modality therapy for rectal cancer in elderly patients aged 75 years and older. Int J Radiat Oncol Biol Phys.

[12] Tougeron D, Roullet B, Paillot B, Hamidou H, Tourani JM, Bensadoun RJ, Michel P, Silvain C (2012). Safety and outcome of chemoradiotherapy in elderly patients with rectal cancer: results from two French tertiary centres. Dig Liver Dis.

[13] Cai X, Wu H, Peng J, Zhu J, Cai S, Cai G, Zhang Z (2013). Tolerability and outcomes of radiotherapy or chemoradiotherapy for rectal cancer in elderly patients aged 70 years and older. Radiat Oncol.

[14] Rutten H, den Dulk M, Lemmens V, Nieuwenhuijzen G, Krijnen P, Jansen-Landheer M, van de Poll Franse L, Coebergh JW, Martijn H, Marijnen C, van de Velde C (2007). Survival of elderly rectal cancer patients not improved: analysis of population based data on the impact of TME surgery. Eur J Cancer.

[15] Kapiteijn E, Marijnen CA, Nagtegaal ID, Putter H, Steup WH, Wiggers T, Rutten HJ, Pahlman L, Glimelius B, van Krieken JH, Leer JW, van de Velde CJ (2001). Preoperative radiotherapy combined with total mesorectal excision for resectable rectal cancer. N Engl J Med.

[16] van Gijn W, Marijnen CA, Nagtegaal ID, Kranenbarg EM, Putter H, Wiggers T, Rutten HJ, Påhlman L, Glimelius B, van de Velde CJ (2011). Preoperative radiotherapy combined with total mesorectal excision for resectable rectal cancer: 12-yearfollow-up of the multicentre, randomised controlled TME trial. Lancet Oncol.

[17] Wakabayashi H, Sano T, Yachida S, Okano K, Izuishi K, Suzuki Y (2007). Validation of risk assessment scoring systems for an audit of elective surgery for gastrointestinal cancer inelderly patients: an audit. Int J Surg.

[18] Ferjani AM, Griffin D, Stallard N, Wong LS (2007). A newly devised scoring system for prediction of mortality in patients with colorectal cancer: a prospective study. Lancet Oncol.

[19] de Vries S, Jeffe DB, Davidson NO, Deshpande AD, Schootman M (2014). Postoperative 30-day mortality in patients undergoing surgery for colorectal cancer: development of a prognostic model using administrative claims data. Cancer Causes Control.

[20] Patel S, Lutz JM, Panchagnula U, Bansal S (2012). Anesthesia and perioperative management of colorectal surgical patients - A clinical review (Part 1). J Anaesthesiol Clin Pharmacol.

[21] Teeuwen PH, Bremers AJ, Groenewoud JM, van Laarhoven CJ, Bleichrodt RP (2011). Predictive value of POSSUM and ACPGBI scoring in mortality and morbidity of colorectal resection: a case-control study. J Gastrointest Surg.

[22] Richards CH, Leitch EF, Horgan PG, Anderson JH, McKee RF, McMillan DC (2010). The relationship between patient physiology, the systemic inflammatory response and survival in patients undergoing curative resection of colorectal cancer. Br J Cancer.

[23] Parsons DP (2009). Preoperative evaluation and risk management. Clin Colon Rectal Surg.

